# Molecular Surface Mesh Generation by Filtering Electron Density Map

**DOI:** 10.1155/2010/923780

**Published:** 2010-04-12

**Authors:** Joachim Giard, Benoît Macq

**Affiliations:** Communications and Remote Sensing Laboratory, Catholic University of Louvain, B-1348 Louvain-la-Neuve, Belgium

## Abstract

Bioinformatics applied to macromolecules are now widely spread and in continuous expansion. In this context, representing external molecular surface such as the Van der Waals Surface or the Solvent Excluded Surface can be useful for several applications. We propose a fast and parameterizable algorithm giving good visual quality meshes representing molecular surfaces. It is obtained by isosurfacing a filtered electron density map. The density map is the result of the maximum of Gaussian functions placed around atom centers. This map is filtered by an ideal low-pass filter applied on the Fourier Transform of the density map. Applying the marching cubes algorithm on the inverse transform provides a mesh representation of the molecular surface.

## 1. Introduction

The geometric structure of macromolecules, such as proteins or nucleic acids, is directly related to their function [1–3]. Consequently, studying this structure is of capital importance in the understanding and simulation of numerous life processes. It allows researchers to save a lot of time and money for various applications such as drug design [4, 5] or mutation effect prediction [6, 7]. In this context, working with molecules external surface can be useful, for instance, to predict the geometrical complementarity between two molecules [8] or to visualize them [9]. The prediction of geometric complementarity is one of the keystones of molecular docking [10–13], the modeling of interactions between molecules. The localization of potential binding sites of molecules [14–16] is a frequently used tool for docking and generally requires a good description of the protein surface [17–19]. The estimation of the surface area may also be related to the stability of a particular molecule 3D conformation [20].

**Figure 1:**
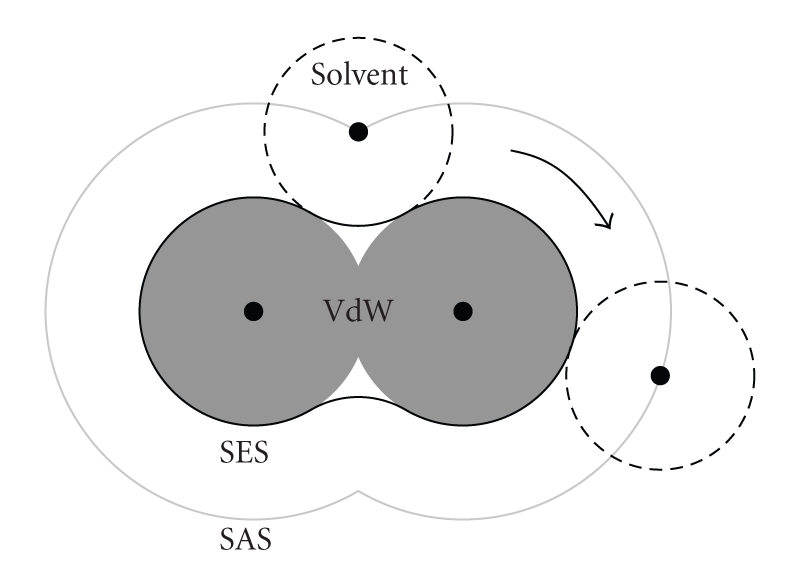
A cutaway view of a small molecule. The most frequent molecular surface representations are detailed. Gray discs depict the Van der Waals volumes, the gray outer line depicts the Solvent Accessible Surface (SAS) and the continuous black line depicts the Solvent Excluded Surface (SES). The SAS (resp., SES) is the limit surface for the solvent molecule center (resp., external surface). The solvent molecule is represented as a black dashed circle.

At first, the external surface of a molecule has to be defined. Indeed, molecules are made of atoms which have no real surface. The most frequent molecular surface representations are the Van der Waals Surface (VdWS), the Solvent Accessible Surface (SAS), and the Solvent Excluded Surface (SES) [21, 22]. In the case of the VdWS, the electron clouds around atoms are approximated by rigid spheres with radii corresponding to the Van der Waals (VdW) radii of the atoms. The SAS (resp., SES) is the inner surface of the volume filled by the possible positions of the center (resp., exterior surface) of a ball representing a molecule of solvent, for example, water (see Figure 1). 

Efficient tools to represent such surfaces are the polygonal meshes, which are collection of points related by edges and faces that approximate the considered surfaces. A lot of methods have been proposed in the last few years for the generation of a molecular surface meshes.

However, the computational time remains generally high for quality meshes, and it can be a problem when there is a great amount of data to treat. In this paper, we introduce the Filtered Density Map (FDM) algorithm, which is a fast and parameterizable algorithm to generate smooth molecular surface meshes. The generated mesh is the isosurface of frequency filtered electron density map.

This paper is organized as follows. First, some other works related to molecular surface generation are succinctly described in Section 2. Then, the FDM method is described in details in Section 3. And finally, results and comparisons with other methods are presented and discussed in Section 4.

## 2. Related Work

In the last few years, a lot of methods have been developed for the generation of molecular surface meshes. In 1983, Connolly [23] proposed an analytical algorithm in which points were strategically placed around the molecule with a specific analytical role (maximum, minimum or saddle point) depending on the number of atoms present in the neighborhood. In 2003, Bajaj et al. [24] introduced another analytical method based on NURBS that offers the advantage to be parameterizable without recalculation. In 2002, Laug and Borouchaki [25] used a parametric representation of intersecting spheres to create the surface mesh. MSMS, developed by Sanner et al. [26] is based on alpha-shapes [27] of molecules. This algorithm is widely used because it is time efficient. However, the generated mesh is not a manifold and is composed of very irregular triangles. The beta-shapes [28] are a generalization of the alpha-shapes and were used by Ryu et al. [29] in 2007 to design a similar algorithm. Another vertex based method was used by Cheng and Shi [30]. In this method, molecular surfaces are generated with the help of restricted union of balls. Finally, some methods based on volumetric computation exist, such as the one of Zhang et al. [31] in which the solvent accessible surface is seen as the isosurface of Gaussian shaped electron density maps, and the algorithm of Can et al. [32] (the LSMS) which is based on a front propagation from atom center and on level-sets.

Comparisons between the FDM method and the methods mentioned in this section are shown in Section 4.2.

## 3. Method

The FDM method is based on volumetric electron density and a frequency filtering. Each atom is seen as a Gaussian electron cloud, the dimensions of which are depending on the VdW radius. Then, the electron density map is created by taking the local maximum value of these clouds. After a Fourier Transform, it is filtered by an ideal low pass filter, in order to remove frequencies corresponding to a spatial element smaller than a solvent molecule. Finally, a marching cubes [33] algorithm is used on the inverse Fourier Transform to find an isosurface. A refinement of the final mesh constitutes an optional step of the method. The whole algorithm was implemented in C++ with vtk (Visual ToolKit) (http://www.vtk.org/).

### 3.1. Electron Density Map

A Gaussian function is constructed around each atom. The value of this function at a point *i* for an atom *a* is:

(1) $$\begin{array}{c} G_{a}\left(i\right)=te^{-(r_{a}^{2}-{||ai||}^{2}/r^{2})}, \end{array}$$

where *t* is a threshold parameter, *r* is a radius parameter, ||*a* *i*|| is the Euclidean distance between the center of *a* and the point *i*, *r*_*a*_ is the VdW radius of the atom *a*. So, the isosurface for the threshold *t* is the VdW sphere because if *i* is located on the VdWS of *a*, ||*a* *i*|| = *r*_*a*_ and *G*_*a*_(*i*) = *t*. In this work, *r* is set to 3 Å because this value is suitable for an ideal low-pass filter (see Section 3.2).

For the implementation, the three-dimensional space is divided into voxels. The spacing (*T*_*e*_) between voxels is an adaptable parameter. The more *T*_*e*_ is small, the more the surface approximation is fine.

The density map of the whole molecule for a point in the space is defined as the maximal value of all the Gaussian functions at this point. The maximum of the Gaussian functions is chosen instead of the summation because it is not possible to evaluate the SES using the isosurface of a summation of Gaussian functions. It can be shown by the following counterexample, in which the Gaussian affected to the atoms *a* and *b* must have contradictory properties depending on the situation.

**Figure 2:**
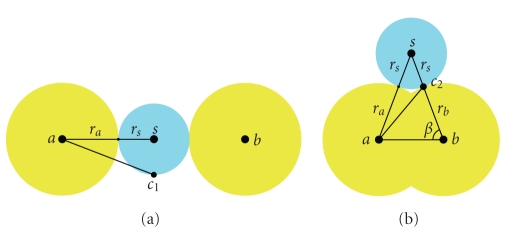
Two situations in which the Gaussian functions would have contradictory properties. (a) Two atoms of the molecule, *a* and *b*, are just close enough to block the way to a solvent molecule *s*. The point *c*_1_ is on the SES and must be influenced by the filed generated by *a*, *G*_*a*_. (b) *a* and *b* are close. The point *c*_2_ is on the SES but also on the VdWS. So, it should not be influenced by *G*_*a*_. It is possible to show that in some (frequent) configurations, ||*a* *c*_1_|| > ||*a* *c*_2_||.

In the first situation, the space between *a* and *b* is just small enough to block the way to a solvent molecule (see Figure 2(a)). The other atoms are considered to be too far to have an influence. Thus, the SES has a concave shape at this place and the value of the density map at the “center” of the concavity *c*_1_ must be influenced by the fields of *a* and *b*: *G*_*a*_(*c*_1_) > 0, *G*_*b*_(*c*_1_) > 0. We can state that ||*a* *c*_1_|| > *r*_*a*_ + *r*_*s*_ because *s*, the center of the solvent molecule, can be very close to the *a* *b* axis. 

In the second situation, ||*a* *b*|| < *r*_*a*_ + *r*_*s*_/2, which is often the case for covalent bonds. Let *c*_2_ be a point belonging both to the SES and to the VdWS of *b* with the necessary condition ||*b* *c*_2_|| = *r*_*b*_ (see Figure 2(b)). The point *c*_2_ should not be influenced by the field of *a*, so *G*_*a*_(*c*_2_) = 0. If ||*a* *c*_2_|| > ||*a* *c*_1_||, this condition is in contradiction with *G*_*a*_(*c*_1_) > 0, because the Gaussian function is strictly decreasing in the positive domain. Using the Al-Kashi theorem, we know that

(2) $$\begin{array}{cc} {||ac_{2}||}^{2} & =r_{b}^{2}+{||ab||}^{2}-2r_{b}||ab||\cos\;\beta ,\\ {||ac_{1}||}^{2}>{\left(r_{a}+r_{s}\right)}^{2} & ={\left(r_{b}+r_{s}\right)}^{2}+{||ab||}^{2}\\ & \;-2\left(r_{b}+r_{s}\right)||ab||\cos\;\beta , \end{array}$$

where *β* is the $\hat{abf}$ angle. Thus, ||*a* *c*_1_|| > ||*a* *c*_2_|| if

(3) $$\begin{array}{c} r_{s}^{2}+2r_{b}r_{s}-2r_{s}||ab||\cos\;\beta >0, \end{array}$$

what is verified by the hypothesis: ||*a* *b*|| < *r*_*b*_ + *r*_*s*_/2 because cos *β* ≤ 1.

In order to avoid interferences, the maximum is preferred to the summation of Gaussian functions. Isosurfacing this density map returns the VdWS. This surface is not smooth and in order to compute the SES, the density map must first be filtered.

### 3.2. Fourier Transform and Filtering

**Figure 3:**
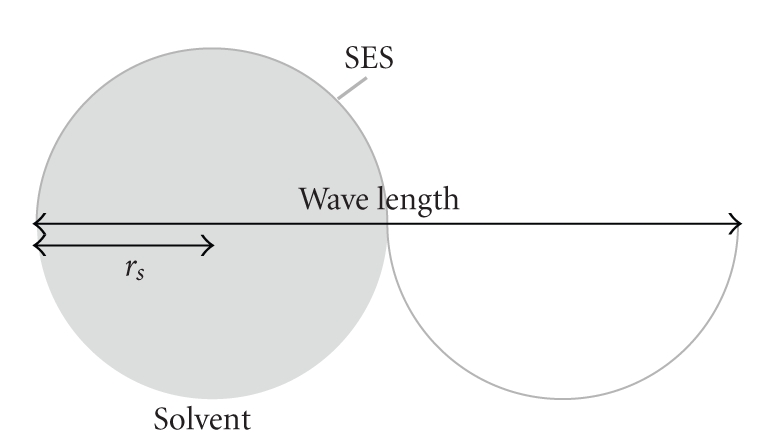
Minimal wavelength allowed on the SES. It corresponds to four times the solvent radius *r*_*s*_ and determines the cutoff frequency for the filtering.

The Fourier Transform of this electron density map is computed using the FFT algorithm [34]. The frequency representation of the function is filtered by an ideal low pass filter in order to eliminate frequencies corresponding to elements smaller than a solvent molecule, for example, inflexion points between two VdW spheres.The cutoff frequency is *f*_*c*_ = 1/4*r*_*s*_, where *r*_*s*_ is the radius of the sphere approximating the solvent molecule (typically 1.4 Å for water). The wavelength must be four times longer than *r*_*s*_ because a molecule solvent diameter has to fit in a half wavelength (see Figure 3). 

Gaussian functions are preferred to balls in the spatial domain because an ideal low-pass filter makes the Gibb's phenomenon appear on sharp edges. An ideal filter is used because the cutoff frequency is exactly known and because it is numerically possible. An ideal low-pass filter in the frequency domain is equivalent to a convolution product with a sinc function in the space domain. Let *X* = (*x*, *y*, *z*) be the space variable in ℝ^3^ and *ℳ*(*X*) the initial electron density map. Then, the filtered density map is:

(4) $$\begin{array}{c} \tilde{ℳ}\left(X\right)=\left(ℳ\left(•\right)*2f_{c}\;\text{sinc}\;\left(2f_{c}•\right)\right)\left(X\right), \end{array}$$

where • represents the variable. The parameter *r* of the Gaussian functions in (1) is related to the width of the function. To keep the isosurface at the same place, a wider function has a smaller maximum. If this maximum is too high, that is, if *r* is too small, the secondary ripples of the sinc function take too much importance when they are in phase with this maximum. It makes oscillations appear in the final density map, which can lead to the apparition of unwanted surfaces after isosurfacing. Simulations with several 1D and 2D functions were performed to verify the effect of *r*. The three main conditions to verify are the follwing:

(5) $$\begin{array}{cc} \tilde{ℳ}\left(X_{c_{1}}\right) & =t\;\text{for}\;\;c_{1}\;\;\text{lying}\;\text{on}\;\text{the}\;\text{SES}\;\text{and}\;\text{on}\;\text{the}\;\text{VdWS},\\ \tilde{ℳ}\left(X_{c_{2}}\right) & =t\;\text{for}\;\;c_{2}\;\;\text{lying}\;\text{on}\;\text{the}\;\text{SES}\;\text{but}\;\text{not}\;\text{on}\;\text{the}\;\text{VdWS},\\ \tilde{ℳ}\left(X_{c_{3}}\right) & <t\;\text{for}\;\;c_{3}\;\;\text{lying}\;\text{outside}\;\text{the}\;\text{molecule}. \end{array}$$

**Figure 4:**
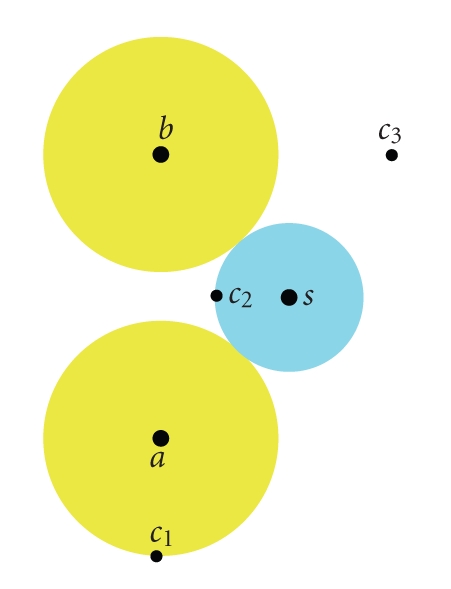
In this example, the atoms *a* and *b* are 5 Å far from each other and have both a VdW radius of 1.8 Å. The radius of the solvent molecule *s* is 1.4 Å. The conditions to verify for the density map before (*ℳ*) and after ($\tilde{ℳ}$) filtering are: $ℳ(c_{1})=\tilde{ℳ}(c_{1})=t$, $ℳ(c_{2})<\tilde{ℳ}(c_{2})=t$, and $ℳ(c_{3})<t\;\text{and}\;\tilde{ℳ}(c_{3})<t$, with *t*, the threshold value for the isosurfacing.

**Figure 5:**
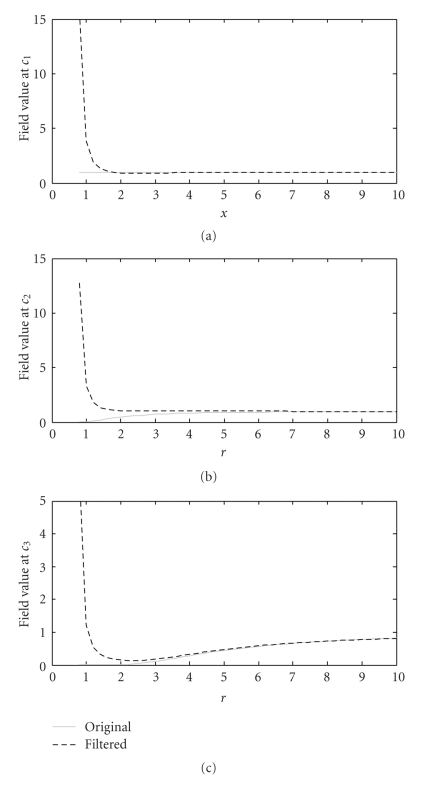
Value of the density map before (continuous gray line) and after (dashed black line) filtering at a point belonging to both the SES and the VdWS, *c*_1_ (a) a point belonging to the SES but not the VdWS, *c*_2_ (b) and a point outside the molecule, *c*_1_ (c) (see Figure 4). *t* = 1, so, when *r* = 3, conditions (5) are satisfied.

**Figure 6:**
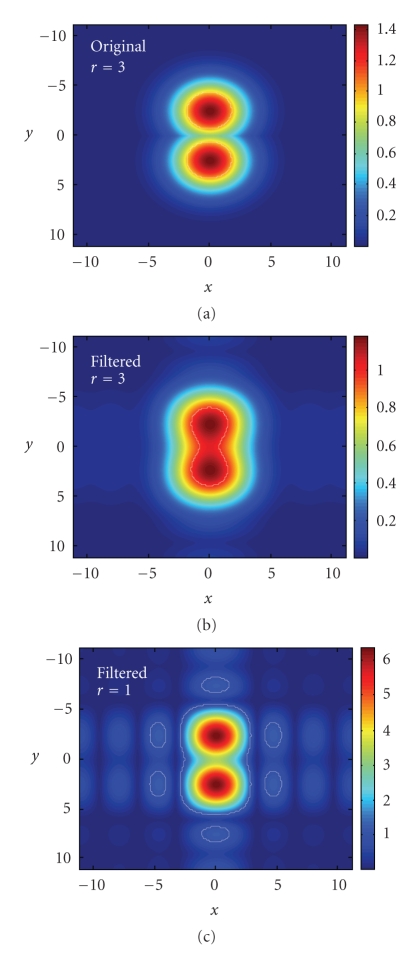
Value of the density map for the example of Figure 4. *x* and *y* are the two spatial dimensions (Å) and the colors represent the value of the density map before filtering and with *r* = 3 (a) after filtering and with *r* = 3 (b) and after filtering with *r* = 1 (c). The contour for *t* = 1 is depicted with a white line. It represents the VdWS (a), the SES (b), and the SES with artifacts (c).

Here is a 2D example:

(6) $$\begin{array}{c} ℳ\left(x\right)=\max\;\left(G_{a}\left(x\right),G_{b}\left(x\right)\right), \end{array}$$

with the atom *a* centered in (*x*_*a*_, *y*_*a*_) = (0, −2.5) and the atom *b* in (*x*_*b*_, *y*_*b*_) = (0,2.5), the threshold *t* = 1, and *r*_*a*_ = *r*_*b*_ = 1.8 (Figure 4). The values of the density maps *ℳ*(*X*) and $\tilde{ℳ}(X)$ for *c*_1_ = (0, *x*_*a*_ − *r*_*a*_), $c_{2}=\left(\sqrt{x_{a}^{2}+{\left(r_{a}+r_{s}\right)}^{2}}-r_{s},0\right)$, and *c*_3_ = (*x*_3_, *y*_3_) are plotted as a function of *r* in Figure 5. (*x*_3_, *y*_3_) is the position of the maximal value of the density map outside the “molecule” for *r* = 1. When the parameter *r* = 3, conditions (5) are verified and the Gaussian functions are not too wide, what leads to shorter execution times. The 2D density maps *ℳ*(*X*) and $\tilde{ℳ}(X)$ with *r* = 3 as well as $\tilde{ℳ}(X)$ with *r* = 1 are shown in Figure 6. The isocontours, representing the VdWS or the SES, are depicted in white and we can see the artifacts appearing for too small values of *r*. 

It is important to notice that if the spacing (*T*_*e*_) for the spatial sampling is too large, there would be no filtering. Normally, the sampling frequency, *f*_*e*_ = 1/*T*_*e*_, has to verify the Nyquist-Shannon theorem: *f*_*e*_ > 2*f*_max_, where *f*_max_ is the higher frequency with a nonzero coefficient in the original signal. However, for this application the errors resulting from a subsampling are not too important and the sampling frequency is chosen such that *f*_*e*_ > 2*f*_*c*_, that is, *T*_*e*_ < 2*r*_*s*_. In this situation, the filtering is always possible.

### 3.3. Isosurfacing

The final triangular mesh is an approximation of the isosurface of the filtered electron density map. The most popular technique to extract an isosurface from a 3D image is the marching cubes algorithm [33]. In this algorithm, the voxels are screened by group of eight sharing a same point. Mesh vertices, faces, and edges are added depending on the value of these eight voxels. There are 256 (2^8^) possibilities that can be reduced to 15 situations thanks to symmetries and complementarities.

### 3.4. Refinement

The visual appearance of the final mesh can be improved by magnifying the number of vertices. The number of vertices is increased using a smooth interpolation scheme such as the piecewise smooth surface reconstruction of Hoppe et al. [35], or the algorithm based on the butterfly scheme proposed by Zorin et al. [36].

## 4. Results and Discussion

 Some numerical results pointing out advantages and drawbacks of the FDM are shown in this section. The main characteristics to be observed are the computation time and the quality of the generated mesh. The section is divided into three parts: the analysis of the effects of the different parameters of the FDM, the results of computation time comparisons with other existing methods, and a quality measurement of the generated meshes.

### 4.1. Parameters

There are three main parameters modifiable by the user. First, the spatial spacing *T*_*e*_, that is, the distance between two neighbor voxels center, which determines the total number of voxels. Second, the cutoff frequency *f*_*c*_, which determines the smoothness of the final mesh. And third, the refinement rate *k*_*r*_, that is, the number of new points in a triangle for the final mesh magnification. In this section, the effect of these parameters on the visual quality, on the computation time and on the memory space, are discussed.

#### 4.1.1. Spatial Spacing

**Figure 7:**
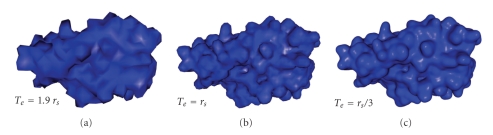
Meshes generated from electron density map at a spatial spacing of *T*_*e*_ = 1.9*r*_*s*_ (a), *T*_*e*_ = *r*_*s*_ (b), and *T*_*e*_ = *r*_*s*_/3 (c) (PDB code: 3EBZ).

With a small spatial spacing, it is possible to represent fine details. However, it drastically increases the memory space needed as well as the computation time. Indeed, reducing *T*_*e*_ by a factor *α* increases the number of voxels by *α* ^3^. The parts of the method depending on the number of voxels are the creation of the density map (time: *𝒪*(*n*_*v*_) and space: *𝒪*(*n*_*v*_), with *n*_*v*_ the number of voxels) and the Fast Fourier Transform (time: *𝒪*(*n*_*v*_log  *n*_*v*_) and space: *𝒪*(*n*_*v*_)). A visual comparison between meshes generated with *T*_*e*_ = 1.9*r*_*s*_, *T*_*e*_ = *r*_*s*_ and *T*_*e*_ = *r*_*s*_/3 is shown in Figure 7. In these examples, *f*_*c*_ = 1/4*r*_*s*_ and *k*_*r*_ = 0, that is, the meshes represent the SES without final refinement.

#### 4.1.2. Cutoff Frequency

**Figure 8:**
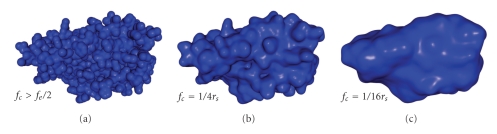
Meshes generated from electron density map filtered at a cutoff frequency of *f*_*c*_ > *f*_*e*_/2 (a) to give the VdWS, *f*_*c*_ = 1/4*r*_*s*_ (b) to give the SES, and *f*_*c*_ = 1/16*r*_*s*_ (c) to give an approximation of the general shape. (PDB code: 3EBZ).

In order to generate a mesh representing the SES, *f*_*c*_ is set to 1/4*r*_*s*_ (see Section 3.2). However, depending on the application, the surface could be other than the SES. For instance, if *f*_*c*_ > *f*_*e*_/2, there is no actual filtering, and so, the generated mesh represents the VdWS. On the other hand, to obtain a smooth approximation of the molecule shape, *f*_*c*_ can be reduced. It is equivalent to consider a bigger solvent molecule. Changing this parameter does not have any effect neither on the computation time nor on the memory space needed. A visual comparison between meshes generated with *f*_*c*_ > *f*_*e*_/2 (VdWS), *f*_*c*_ = 1/4*r*_*s*_, and *f*_*c*_ = 1/16*r*_*s*_ (shape approximation) is shown in Figure 8. In these examples, *T*_*e*_ = *r*_*s*_/3 and *k*_*r*_ = 0, that is, a solvent molecule diameter takes 6 voxels and there is no final refinement.

#### 4.1.3. Refinement Factor

**Figure 9:**
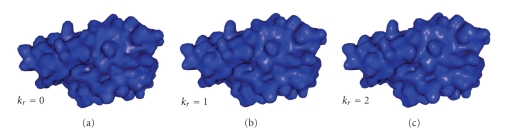
Meshes refined with a factor of *k*_*r*_ = 0 (a), *k*_*r*_ = 1 (b), and *k*_*r*_ = 2 (c) (PDB code: 3EBZ).

The final mesh refinement gives foremost an esthetic advantage. The memory space needed does not increase a lot because the number of voxels remains the same. Only the size of the mesh changes and this is negligible in comparison with the space needed by the voxels representation. The computation time slightly increases but, when *k*_*r*_ = 1 (which gives a good visual improving), this is negligible in comparison with the voxels operations computation time. A visual comparison between meshes generated with *k*_*r*_ = 0, *k*_*r*_ = 1 and *k*_*r*_ = 2 is shown in Figure 9. In these examples, *T*_*e*_ = *r*_*s*_ and *f*_*c*_ = 1/4*r*_*s*_, that is, a solvent molecule diameter takes 2 voxels and the meshes represent the SES.

### 4.2. Time Comparisons

**Figure 10:**
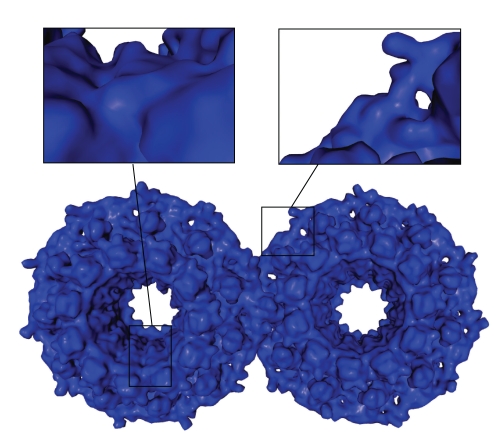
Mesh of the SES of a molecule with *T*_*e*_ = 4/3*r*_*s*_ and *k*_*r*_ = 1. (PDB Code: 1H2I).

In this section, computation times are compared between the FDM algorithm and algorithms found in the literature for equivalent qualities. When available, the algorithms were run on the same computer, when not, the computation times were the ones announced in the original paper. Can et al. made a comparison of their method computation time with three molecular visualization tools: UCSF Chimera [37], Swiss-PDBViewer [38], and PyMol [9]. We added MSMS [26] to this set of methods. These programs, as well as the one of Can (LSMS), are available for free, so, the computation time could be measured on the same computer than for our method, except for Chimera that was not supported by the system. Thus, the computation times mentioned here for Chimera are the one announced in the paper of Can et al. [32]. For the LSMS method, the grid size is set to 256 × 256 × 256. In this condition, *T*_*e*_ ≃ 1 with the tested molecules. Other programs are run with defaults settings, that is, *T*_*e*_ ≃ 1. Two tests are made for the FDM method. In the first one, the parameters are set to *T*_*e*_ = 1.9*r*_*s*_ and *k*_*r*_ = 0 to be as fast as possible while keeping a correct solution. In the second one, the parameters are set to *T*_*e*_ = 4/3*r*_*s*_ and *k*_*r*_ = 1 which gives a correct mesh with a good appearance (see Figure 10). The computation times are shown in Table 1. The computation times reported only include the mesh generation time, that is, it does not take the loading time into account. In addition, for three molecules, computation times for the method of Cheng and Shi [30] are reported from their paper. The computation times comparison is shown in Table 2. All the tests were performed on a AMD Athlon(tm) 64 X2 Dual Core Processor 3800+, 2 gigabytes RAM. The computers were more or less equivalent in the cited papers.

It appears in Tables 1 and 2 that all the molecule surfaces in this data set are computed faster with the FDM than with any other method and for different values of the parameters.

### 4.3. Quality Results

In order to validate the quality of the results, different generated surfaces (SESs) were compared with references surfaces. These reference surfaces were generated by isosurfacing a field composed of a union of VdW balls at good resolution (spatial spacing of *r*_*s*_/8) after morphological closing with a structuring element of the size of the solvent molecule. This approach, similar to [32], directly follows the definition of the SES [22], because morphologically close this volume is equivalent to make a solvent molecule roll on the VdW balls and to consider unaccessible parts to be inside the molecule. The molecules tested were 200D, 1FG1, and 3EBZ, because they are small enough to generate a good reference surface with the available memory space. The mean weighted root mean square deviation (RMSD) for three spatial spacing is reported in Table 3. The weighted RMSD is

(7) $$\begin{array}{c} {\text{RMSD}}_{w}=\sqrt{\frac{\sum_{n=1}^{N}{||p_{n}p_{n}^{\prime }||}^{2}s_{n}}{S}}, \end{array}$$

where *N* is the number of vertices in the reference mesh, *p*_*n*_ is a vertex of this mesh, *p*_*n*_′ is the closest point on the other mesh (not necessary a vertex, it can be on an edge or on a face), *s*_*n*_ is the mean area of the faces *p*_*n*_ belongs to, and *S* is the total surface area. The percentages of the surface for which ||*p*_*n*_ *p*_*n*_′|| is greater than the spacings are shown in the right-hand-side columns of Table 3. These error indexes are not completely correct because the reference surfaces is not a ground truth. However, it shows that the FDM algorithm can provide a surface with a quality comparable to robust methods.

**Figure 11:**
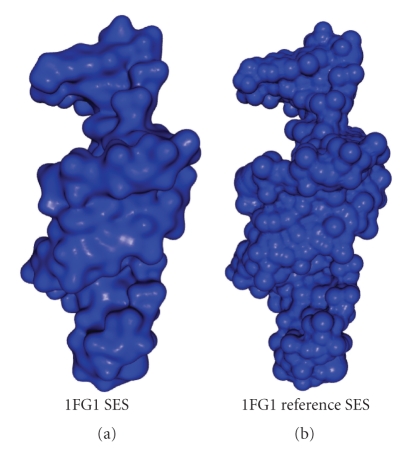
Visual comparison between the 1FG1 SES generated with the FDM algorithm with a spacing of *r*_*s*_/4 and the reference SES generated with a spacing of *r*_*s*_/8.

A visual comparison between the SES of 1FG1 computed with the FDM algorithm and the reference SES is shown in Figure 11. 

## 5. Conclusion

In this paper, we introduced an algorithm to compute molecular surface meshes (the FDM algorithm). It is constructed as an isosurface of a filtered electron density map (FDM). This algorithm is faster than other algorithm tested in equivalent conditions. It is slower than the MSMS algorithm for small molecules (<30000 atoms) but it returns a smooth manifold surface, which is not the case with MSMS. It makes possible to compute a precise representation of the surface with a limited number of voxels, so that the computation time and the memory space needed are reduced. Moreover, it is parameterizable on the spatial resolution, the refinement of the final mesh, and the size of the solvent molecule. Thus, the spatial resolution can be improved for a finer result but with an important computation time increase. Similarly, a smoother result can be obtained with a final refinement with a small influence on the computation time but with less precise results than reducing the spacing. Finally, the solvent molecule size can be chosen without influence on the computation time.

The refinement could be improved to be specific to molecular surface. It would enable coarse meshes to be generated rapidly and to be improved by a priori knowledge about local geometry of molecule surfaces, such that the curvature deduced from the closest atom radius. In future works, this algorithm will be used in surface-based method to detect protein hot spots [19].

## Figures and Tables

**Table 1:** Computation times (in s) for different methods. (^(*b*)^ from [32].)

PDB code	No. atoms	FDM_1.9*r*_*s*_,0_	FDM_4/3*r*_*s*_,1_	LSMS	PyMol	Swiss-PDBV	Chimera	MSMS
1A8R	26400	0.65	2.61	5.56	10.52	6.38	16.36^(**b**)^	0.95
1H2I	32318	0.72	1.83	6.50	11.37	5.25	40.04^(**b**)^	3.03
1GTP	34740	0.51	2.20	6.98	13.15	4.75	67.04^(**b**)^	9.02
1FKA	34977	0.75	2.77	7.89	26.29	7.36	77.25^(**b**)^	4.50
1GT7	42700	0.95	2.43	7.32	16.10	6.50	54.39^(**b**)^	3.32
1GAV	43335	0.55	2.54	7.05	28.86	7.71	78.35^(**b**)^	4.22
1G3I	45528	0.54	2.85	8.18	19.45	6.21	—	7.67
1PMA	45892	0.40	1.97	8.23	18.67	6.72	—	12.90
1FJG	51995	0.71	2.88	8.01	25.18	8.05	—	15.11
1AON	58870	0.62	2.64	8.83	26.36	8.91	—	10.87
1J0B	60144	0.69	3.07	6.87	32.66	7.92	—	5.61
1OTZ	68620	0.67	2.28	8.46	30.14	9.56	—	9.03
1IR2	77088	0.65	2.88	7.09	29.31	9.55	93.87^(**b**)^	9.49

**Table 2:** Computation times (in s) for different methods. (^(*a*)^ from [30].)

PDB code	No. atoms	≃ No. triangles	FDM_*r*_*s*_/2,1_	Cheng	MSMS
200D	232	65 k	0.55	1.35^(**a**)^	0.33
1FG1	873	100 k	0.85	2.41^(**a**)^	0.65
3EBZ	1651	200 k	1.37	15.43^(**a**)^	0.97

**Table 3:** RMSD and percentage of big differences with reference surfaces.

Spacing (Å)	RMSD_*w*_ (Å)	||·|| > 1.9*r*_*s*_	||·|| > *r*_*s*_	||·|| > *r*_*s*_/4
1.9*r*_*s*_ = 2.66	0.78	**0.41%**	6.89%	67.04%
*r*_*s*_ = 1.4	0.26	0%	**0%**	20.20%
*r*_*s*_/4 = 0.35	0.20	0%	0%	**8.71%**
